# ASC-Derived Extracellular Vesicles Suppress Macrophage-Driven Inflammatory Amplification and Contractile Activation of Uterine Smooth Muscle Cells

**DOI:** 10.3390/ijms27104273

**Published:** 2026-05-11

**Authors:** Ji-Seon Lee, You-rin Kim, Dogeon Yoon, Ji Hye Park, Tae-Keun Kim, Eun-Kyoung Choi, Jun Hur, Ji-Eun Song

**Affiliations:** 1 Burn Institute, Hangang Sacred Heart Hospital, College of Medicine, Hallym University, Seoul 07247, Republic of Korea; 2 Ilsong Institute of Life Science, Hallym University, Seoul 07247, Republic of Korea; dase80@hallym.ac.kr (T.-K.K.); ekchoi@hallym.ac.kr (E.-K.C.); 3 Department of Biomedical Gerontology, Graduate School of Hallym University, Chuncheon 24252, Republic of Korea; 4 Department of Surgery and Critical Care, Burn Center, Hangang Sacred Heart Hospital, Hallym University Medical Center, Seoul 07247, Republic of Korea; 5 Department of Obstetrics and Gynecology, Kangnam Sacred Heart Hospital, Hallym University, Seoul 07441, Republic of Korea

**Keywords:** preterm labor, uterine smooth muscle cells, ASC-EVs, lipopolysaccharide (LPS), NF-κB signaling, MAPK pathway, TLR4 signaling, inflammation, calcium signaling

## Abstract

Preterm labor is a major cause of neonatal morbidity and mortality and is frequently driven by infection-associated inflammation that promotes premature uterine activation. In this study, we investigated the effects of adipose stem cell-derived extracellular vesicles (ASC-EVs) on macrophage-mediated inflammatory signaling in uterine smooth muscle cells (HUtSMCs). An in vitro model was established by treating HUtSMCs with conditioned media derived from LPS-stimulated RAW264.7 macrophages. Activation of signaling pathways was assessed by Western blotting and immunofluorescence, and functional responses were evaluated using calcium flux and collagen gel contraction assays. Conditioned media from LPS-stimulated macrophages induced robust activation of MAPK (ERK1/2 and JNK) and NF-κB signaling, accompanied by IκB degradation and nuclear translocation of phosphorylated p65, whereas ASC-EVs pretreatment significantly attenuated these responses and reduced the expression of pro-inflammatory cytokines, including IL-6, IL-8, and MCP-1. Furthermore, macrophage-conditioned media enhanced intracellular calcium flux and contractile activity in HUtSMCs, both of which were suppressed by ASC-EVs. Inhibition of TLR4 signaling in macrophages reduced the inflammatory potency of conditioned media, indicating a key upstream role of macrophage TLR4 activation. Collectively, these findings demonstrate that ASC-EVs suppress macrophage-mediated inflammatory activation and downstream contractile responses, suggesting their potential as a cell-free therapeutic strategy for preventing inflammation-associated preterm labor.

## 1. Introduction

Preterm labor, defined as childbirth occurring before 37 completed weeks of gestation, remains a major cause of neonatal morbidity and mortality worldwide [1]. It is a multifactorial condition with diverse etiologies, including intrauterine infection, sterile inflammation, uteroplacental ischemia, cervical insufficiency, and maternal or fetal stress. Among these, inflammatory processes are considered key drivers of premature uterine activation [2,3,4].

In infection-associated preterm labor, pathogen-associated molecular patterns (PAMPs), such as lipopolysaccharide (LPS), are conserved microbial components that are recognized by pattern recognition receptors (PRRs) on immune cells, leading to activation of inflammatory signaling pathways [5,6]. In contrast, sterile inflammation is mediated by damage-associated molecular patterns (DAMPs) released from stressed or injured tissues. Both mechanisms contribute to elevated levels of pro-inflammatory cytokines and chemokines—including IL-6, IL-8, and MCP-1—at the maternal–fetal interface, which comprises the decidua, placenta, and fetal membranes, thereby promoting uterine smooth muscle cell (SMC) contraction and accelerating the onset of labor [7,8,9].

Macrophages play a central role in amplifying inflammatory responses during preterm labor [10]. Increased macrophage infiltration has been observed in decidual and myometrial tissues under inflammatory conditions, and activated macrophages release cytokines, chemokines, and other soluble mediators that enhance local inflammation [11,12]. These macrophage-derived signals act on uterine smooth muscle cells, promoting contractility and contributing to uterine activation [10,11,12,13]. However, the mechanisms underlying macrophage–myometrial cell interactions, particularly under inflammatory conditions, remain incompletely understood.

Human uterine smooth muscle cells (HUtSMCs) are key effectors of uterine contractility and are highly responsive to inflammatory stimuli [14,15]. Activation of Toll-like receptor 4 (TLR4) signaling in HUtSMCs induces downstream MAPK (ERK1/2 and JNK) and NF-κB pathways, leading to the production of pro-inflammatory mediators and increased myometrial excitability. Nevertheless, direct stimulation of HUtSMCs with LPS alone does not fully recapitulate the complex in vivo inflammatory environment, where immune cell-derived mediators play a critical role. To better mimic this complex in vivo inflammatory microenvironment in a controlled setting, we employed an in vitro model incorporating macrophage-derived conditioned media. In this context, macrophage-conditioned media generated from LPS-activated macrophages provides a more physiologically relevant model of paracrine inflammatory signaling.

Despite its clinical burden, therapeutic strategies for preterm labor remain limited and largely symptomatic. Current interventions, including tocolytics, progesterone supplementation, and corticosteroids, primarily aim to delay delivery or reduce neonatal complications rather than targeting the underlying inflammatory mechanisms [16,17,18,19]. Therefore, there is a need for novel therapeutic approaches that can effectively modulate both inflammatory and contractile pathways.

Mesenchymal stem cell-derived EVs have emerged as promising cell-free therapeutic agents due to their anti-inflammatory and immunomodulatory properties [20,21,22,23]. Among the various cellular sources of EVs, adipose-derived stem cells (ASCs) are particularly attractive due to their abundance, ease of isolation, and high EVs yield. EVs derived from ASCs (ASC-EVs) have demonstrated beneficial effects in various inflammatory conditions [24,25,26,27,28]. However, their role in regulating macrophage-driven inflammation and uterine contractility remains unclear.

Therefore, this study aimed to investigate whether ASC-derived EVs can suppress macrophage-mediated inflammatory amplification and contractile activation in uterine smooth muscle cells under inflammatory conditions.

## 2. Results

### 2.1. Characterization and Cellular Localization of ASC-Derived Extracellular Vesicles

**Figure 1:**
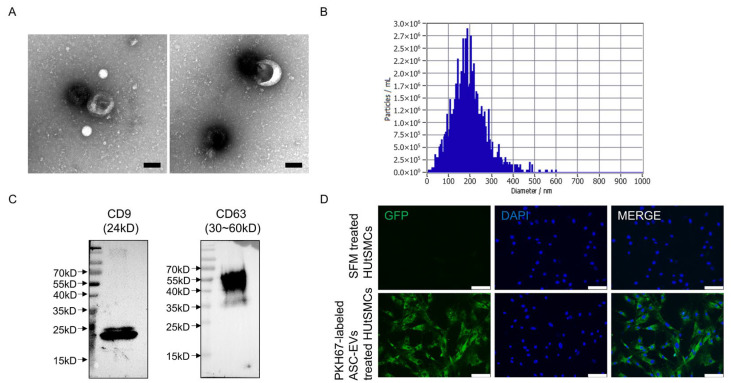
Characterization and cellular localization of ASC-derived EVs. (**A**) Transmission electron microscopy (TEM) images showing vesicles with sizes consistent with extracellular vesicles (Scale bars = 100 nm). (**B**) Size distribution of EVs measured by nanoparticle tracking analysis (NTA), with a median diameter of 183.1 nm and a particle concentration of 3.8 × 10^10^ particles/mL. (**C**) Western blot analysis confirming the expression of EV-associated markers CD9 and CD63. (**D**) Fluorescence microscopy images showing PKH67-labeled EV signals (green) in HUtSMCs. Nuclei were stained with DAPI (blue). Scale bars = 100 mm.

To investigate the biological effects of adipose-derived stem cell-derived EVs (ASC-EVs), EVs were first isolated and characterized. Transmission electron microscopy (TEM) revealed vesicles with a size distribution consistent with extracellular vesicles (Figure 1A). Nanoparticle tracking analysis (NTA) showed that the majority of particles ranged from 100 to 300 nm in diameter, with a median diameter of 183.1 nm and a particle concentration of 3.8 × 10^10^ particles/mL (Figure 1B). Western blot analysis confirmed the presence of EV-associated markers CD9 and CD63 (Figure 1C).

To assess cellular localization of ASC-Derived Extracellular Vesicles, PKH67-labeled ASC-EVs were incubated with HUtSMCs. Fluorescence microscopy demonstrated green fluorescence signals in HUtSMCs, suggesting association of ASC-derived EVs with the cells (Figure 1D).

### 2.2. ASC-Derived EVs Suppress LPS-Induced Inflammatory Signaling in HUtSMCs

**Figure 2:**
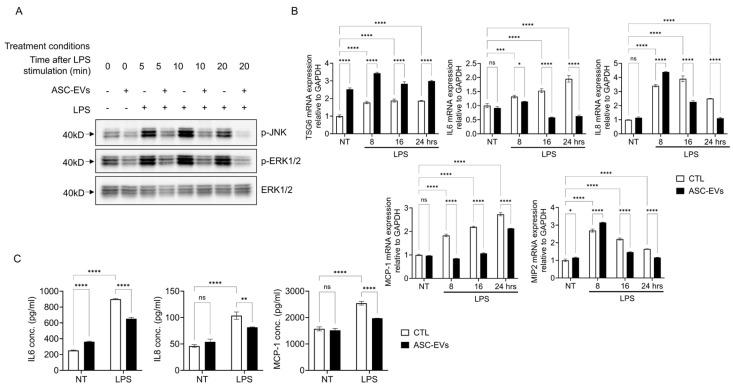
ASC-derived EVs suppress LPS-induced inflammatory signaling in HUtSMCs. (**A**) HUtSMCs were pretreated with ASC-derived EVs and stimulated with LPS for the indicated time points (5–20 min). Phosphorylation of JNK and ERK1/2 was analyzed by Western blotting. (**B**) mRNA expression levels of TSG6, IL-6, IL-8, MCP-1, and MIP-2 were measured by quantitative RT-PCR at 8–24 h after LPS stimulation and normalized to GAPDH. (**C**) Secretion levels of IL-6, IL-8, and MCP-1 were quantified by ELISA. Data are presented as mean ± SEM. * *p* < 0.05, ** *p* < 0.01, *** *p* < 0.001, **** *p* < 0.0001; ns, not significant.

To determine whether ASC-EVs exert protective and anti-inflammatory effects on inflammatory signaling, HUtSMCs were pretreated with ASC-EVs prior to LPS stimulation. Phosphorylation of JNK and ERK1/2, a well-established indicator of MAPK pathway activation, was rapidly induced following LPS stimulation. ASC-EV pretreatment was associated with a reduction in LPS-induced phosphorylation of JNK and ERK1/2, suggesting attenuation of MAPK signaling (Figure 2A).

Quantitative RT-PCR analysis revealed that LPS significantly increased the expression of IL-6, IL-8, MCP-1, and MIP-2 in a time-dependent manner, while ASC-EVs pretreatment reduced their expression (Figure 2B). In contrast, expression of the anti-inflammatory gene TSG6 was increased following ASC-EV treatment.

Consistently, ELISA analysis demonstrated that ASC-EVs significantly reduced LPS-induced secretion of IL-6, IL-8, and MCP-1 (Figure 2C). Cytokine array analysis further confirmed that LPS stimulation markedly increased the secretion of multiple inflammatory mediators, including GRO a/b/g, GRO α, IL-6 and IL-8 (Supplementary Figure S1). Among these, pretreatment with ASC EVs significantly attenuated the LPS-induced upregulation of IL-6, and partially reduced GRO a/b/g and IL-8 expression. These findings suggest that ASC-EVs selectively modulate inflammatory cytokine production under LPS-induced conditions.

### 2.3. ASC-Derived EVs Modulate TLR4-Dependent Inflammatory Signaling

**Figure 3:**
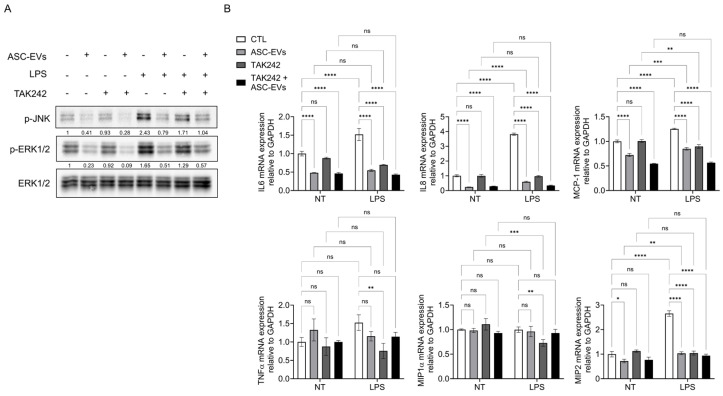
ASC-derived EVs modulate TLR4-dependent inflammatory signaling in HUtSMCs. (**A**) HUtSMCs were pretreated with ASC-EVs and/or a TLR4 inhibitor prior to LPS stimulation. Phosphorylation of JNK and ERK1/2 was analyzed by Western blotting. Representative images from three independent experiments are shown. (**B**) mRNA expression levels of IL-6, IL-8, MCP-1, TNF-α, MIP-1α, and MIP-2 were analyzed by quantitative RT-PCR and normalized to GAPDH. Data are presented as mean ± SEM from three independent experiments and are expressed relative to the untreated control. * *p* < 0.05, ** *p* < 0.01, *** *p* < 0.001, **** *p* < 0.0001; ns, not significant.

To examine whether ASC-EVs modulate TLR4-mediated signaling, HUtSMCs were treated with TAK242, a selective TLR4 inhibitor, in the presence or absence of ASC EVs prior to LPS stimulation. LPS-induced phosphorylation of JNK and ERK1/2 was significantly reduced by both TAK-242 treatment and ASC-EVs pretreatment (Figure 3A), indicating that ASC-EVs may attenuate MAPK activation, at least in part, through modulation of TLR4 signaling (Figure 3A).

Similarly, LPS-induced expression of IL-6, IL-8, MCP-1, and MIP-2 was markedly decreased by both treatments (Figure 3B). No strong additive effect was observed with combined treatment, suggesting that ASC-EVs act, at least in part, through the TLR4 signaling axis. MIP-1α expression remained largely unchanged, indicating selective regulation of inflammatory responses.

### 2.4. ASC-Derived EVs Suppress Macrophage-Amplified Inflammatory Signaling

**Figure 4:**
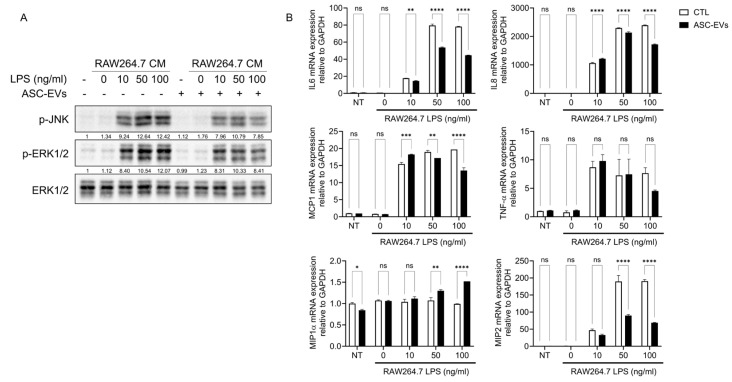
ASC-derived EVs attenuate macrophage-mediated inflammatory signaling in HUtSMCs. (**A**) HUtSMCs were pretreated with ASC-derived EVs and stimulated with conditioned media (CM) derived from RAW264.7 macrophages treated with LPS (10–100 ng/mL). Cells were harvested after 10 min, and phosphorylation of JNK and ERK1/2 was analyzed by Western blotting. (**B**) mRNA expression levels of IL-6, IL-8, MCP-1, TNF-α, MIP-1α, and MIP-2 were analyzed by quantitative RT-PCR 20 h after CM stimulation. Data are presented as mean ± SEM. * *p* < 0.05, ** *p* < 0.01, *** *p* < 0.001, **** *p* < 0.0001; ns, not significant.

To model macrophage-mediated inflammatory amplification, HUtSMCs were treated with conditioned media (CM) derived from RAW264.7 macrophages stimulated with LPS. CM from LPS-stimulated macrophages induced robust phosphorylation of JNK and ERK1/2, whereas CM from unstimulated macrophages had minimal effects (Figure 4A).

ASC-EVs pretreatment significantly attenuated CM-induced MAPK activation. Consistently, CM from LPS-stimulated macrophages markedly increased the expression of IL-6, IL-8, MCP-1, TNF-α, and MIP-2, whereas ASC-EVs significantly reduced these responses (Figure 4B). MIP-1α expression was significantly increased under certain conditions, although the overall changes in response to LPS stimulation and ASC-EV treatment were relatively modest.

### 2.5. ASC-Derived EVs Inhibit Macrophage TLR4-Dependent NF-κB Activation and p65 Nuclear Translocation

**Figure 5:**
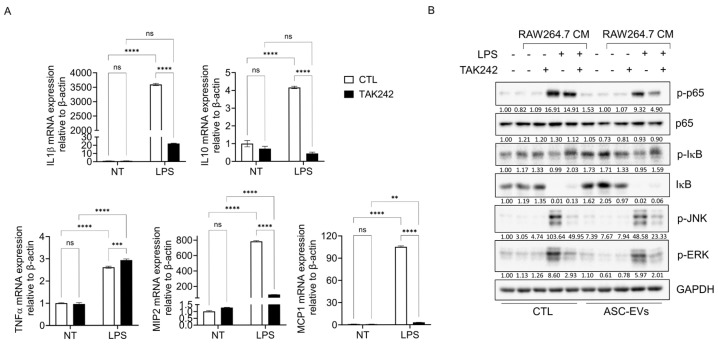

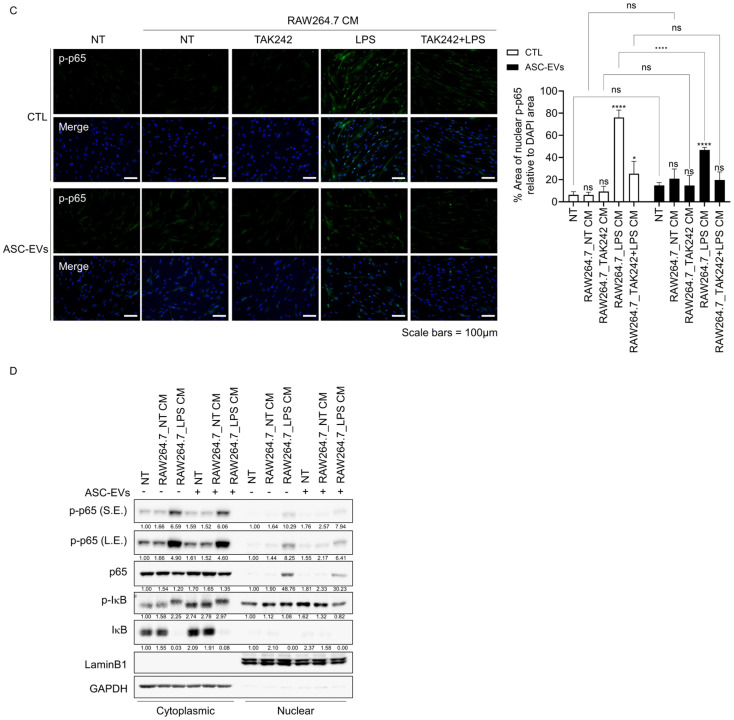
ASC-derived EVs inhibit macrophage TLR4-dependent NF-κB and MAPK activation in HUtSMCs. (**A**) RAW264.7 macrophages were stimulated with LPS in the presence or absence of the TLR4 inhibitor TAK242. mRNA expression levels of IL-1β, IL-10, TNF-α, MIP-2, and MCP-1 were analyzed by quantitative RT-PCR and normalized to β-actin. (**B**) HUtSMCs were treated with conditioned media derived from RAW264.7 macrophages under the indicated conditions, with or without ASC-EVs pretreatment. Phosphorylation of p65, JNK, and ERK1/2 was analyzed by Western blotting. (**C**) Immunofluorescence analysis of phosphorylated p65 (green) in HUtSMCs. Nuclei were stained with DAPI (blue). (**D**) Cytoplasmic and nuclear fractionation analysis of NF-κB signaling. Levels of phosphorylated p65, total p65, phosphorylated IκB, and total IκB were analyzed by Western blotting. α-tubulin and Lamin B1 were used as cytoplasmic and nuclear markers, respectively. Data are presented as mean ± SEM. * *p* < 0.05, ** *p* < 0.01, *** *p* < 0.001, **** *p* < 0.0001; ns, not significant.

To determine whether macrophage-mediated signaling depends on TLR4, RAW264.7 macrophages were stimulated with LPS in the presence or absence of TAK242. LPS stimulation significantly increased the expression of IL-1β, IL-10, TNF-α, MIP-2, and MCP-1. Among these, the expression levels of IL-1β, IL-10, MIP-2, and MCP-1 were markedly reduced by treatment with the TLR4 inhibitor TAK242. In contrast, TNF-α expression showed a moderate increase in response to TAK242 treatment (Figure 5A).

Conditioned media (CM) derived from LPS-stimulated macrophages induced phosphorylation of p65, JNK, and ERK1/2 in HUtSMCs. ASC-EVs pretreatment was associated with a reduction in these phosphorylation events. However, when CM derived from macrophages pretreated with TAK242 prior to LPS stimulation was used, ASC-EV pretreatment had minimal additional effects. These findings suggest that the inhibitory effects of ASC-EVs on LPS-induced signaling are, at least in part, dependent on TLR4-mediated pathways (Figure 5B).

Immunofluorescence analysis revealed that CM from LPS-stimulated macrophages induced nuclear accumulation of phosphorylated p65, whereas this effect was reduced by TLR4 inhibition and ASC-EVs pretreatment (Figure 5C). In contrast, direct LPS stimulation alone did not induce detectable nuclear translocation of phosphorylated p65 in HUtSMCs (Supplementary Figure S2).

Cytoplasmic and nuclear fractionation further showed that CM derived from LPS-stimulated macrophages induced IκB phosphorylation and degradation, accompanied by increased nuclear levels of phosphorylated p65. ASC-EV pretreatment was associated with a modest reduction in nuclear p-p65 levels in the nuclear fraction (Figure 5D).

### 2.6. ASC-Derived EVs Attenuate Macrophage-Mediated Calcium Signaling and Contractile Activity

**Figure 6:**
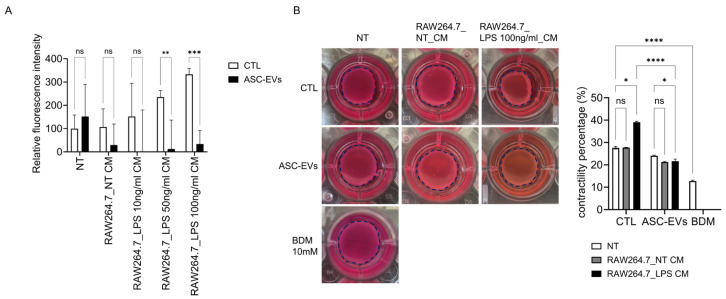
ASC-derived EVs attenuate macrophage-mediated calcium signaling and contractile activity in HUtSMCs. (**A**) Intracellular calcium flux was measured in HUtSMCs following stimulation with conditioned media derived from RAW264.7 macrophages under the indicated conditions. Relative fluorescence intensity was quantified. (**B**) Collagen gel contraction assay of HUtSMCs treated with macrophage-conditioned media with or without ASC-EVs pretreatment. Representative images and quantitative analysis of gel contraction are shown. Blebbistatin (BDM) was used as a positive control. Data are presented as mean ± SEM. * *p* < 0.05, ** *p* < 0.01, *** *p* < 0.001, **** *p* < 0.0001; ns, not significant.

To assess the functional consequences of inflammatory signaling on uterine contractility, intracellular calcium flux—an essential mediator of smooth muscle contraction—was measured. CM from LPS-stimulated macrophages significantly increased intracellular calcium levels, whereas ASC-EVs pretreatment reduced this response (Figure 6A).

To further evaluate contractile function, collagen gel contraction assays were performed. CM from LPS-stimulated macrophages induced strong contraction of HUtSMCs, which was significantly attenuated by ASC-EVs (Figure 6B). Blebbistatin (BDM) nearly abolished contraction, confirming actomyosin-dependent contractility. Direct LPS stimulation induced weaker contraction compared to macrophage CM (Supplementary Figure S3).

## 3. Discussion

Preterm labor is a major cause of neonatal morbidity and mortality, and infection-associated inflammation is widely recognized as a key trigger of premature uterine activation [1]. In the present study, we demonstrate that ASC-derived EVs suppress inflammatory signaling and contractile activation in uterine smooth muscle cells by targeting macrophage-driven inflammatory amplification. These findings provide mechanistic insight into how immune cell-derived signals regulate uterine activation and support the potential of ASC-EVs as a cell-free therapeutic strategy for inflammation-associated preterm labor.

A central finding of this study is that direct LPS stimulation induces relatively limited NF-κB activation in HUtSMCs, whereas conditioned media from LPS-stimulated macrophages elicits robust activation of MAPK and NF-κB signaling pathways. Previous studies have shown that uterine tissues are highly influenced by immune cell-derived cytokines and chemokines during infection-associated preterm labor [29,30,31]. Our data extend these observations by demonstrating that macrophage-derived paracrine signals, rather than direct microbial stimulation, serve as potent drivers of uterine smooth muscle activation. This supports the concept that macrophage-mediated inflammatory amplification represents a critical upstream mechanism in the pathogenesis of preterm labor.

Consistent with previous reports identifying TLR4 as a key sensor of LPS [32,33,34], our results show that macrophage activation is largely dependent on TLR4 signaling. Pharmacological inhibition of TLR4 significantly reduced the inflammatory potency of conditioned media derived from LPS-stimulated macrophages, indicating that TLR4 activation is a major upstream regulator of macrophage-derived inflammatory signals. These signals subsequently activate MAPK and NF-κB pathways in HUtSMCs, linking innate immune activation to downstream uterine contractile responses [35,36].

Mechanistically, ASC-EVs inhibited both early MAPK activation and subsequent NF-κB signaling, suggesting coordinated suppression of inflammatory signaling cascades. Our fractionation and immunofluorescence analyses further revealed that ASC-EVs prevent IκB phosphorylation and degradation, thereby blocking nuclear translocation of phosphorylated p65. Given that p65 phosphorylation enhances transcriptional activity of NF-κB, these findings indicate that ASC-EVs regulate NF-κB signaling at multiple levels, including upstream kinase activation and nuclear translocation. This mechanistic insight expands upon previous studies reporting the anti-inflammatory properties of mesenchymal stem cell-derived EVs and provides direct evidence of their role in modulating NF-κB signaling dynamics. However, it should be noted that the observed inhibitory effects of ASC-derived EVs may not solely reflect direct modulation of intracellular signaling pathways [37,38,39]. It is possible that physical interactions at the cell surface, such as steric hindrance, could partially interfere with ligand–receptor binding, including LPS–TLR4 interactions. Therefore, these mechanistic interpretations should be considered with caution. In addition, further studies using appropriate control particles, such as irrelevant EVs or inert nanoparticles, will be necessary to more clearly distinguish between physical and signaling-mediated effects.

Importantly, suppression of inflammatory signaling by ASC-EVs translated into functional outcomes. Conditioned media from LPS-stimulated macrophages significantly increased intracellular calcium flux and collagen gel contraction in HUtSMCs, both of which are key determinants of uterine contractility. ASC-EVs pretreatment effectively attenuated these responses, demonstrating that modulation of inflammatory signaling directly influences uterine smooth muscle function. These findings are consistent with previous reports linking inflammatory signaling to increased myometrial excitability and further highlight the functional relevance of macrophage-mediated inflammatory pathways in preterm labor.

ASC-derived EVs have been widely reported to exert anti-inflammatory and tissue-protective effects through the delivery of bioactive cargo, including microRNAs, proteins, and lipids. Although the specific molecular components responsible for the observed effects were not identified in this study, our findings suggest that ASC-EVs modulate the TLR4–MAPK–NF-κB signaling axis. Future studies should focus on identifying the key EV cargo responsible for these effects and elucidating their mechanisms of action. In addition, validation in in vivo models of preterm labor will be essential to further establish the therapeutic potential of ASC-EVs.

While the present study provides important insights into macrophage-mediated inflammatory signaling, several limitations should be considered. In particular, we employed a conditioned media-based model to investigate the effects of macrophage-derived soluble factors on uterine smooth muscle cells. While this approach allows us to examine the effects of soluble factors in a controlled setting, it does not fully represent direct cell–cell interactions or two-way communication between macrophages and uterine smooth muscle cells. In the in vivo environment, these cell types coexist and dynamically interact through both paracrine signaling and direct contact-dependent mechanisms. Therefore, future studies using co-culture systems, such as Transwell models, or in vivo approaches will be necessary to more comprehensively understand the complex cellular interactions underlying inflammation-associated preterm labor.

In addition, conditioned media may contain extracellular vesicles and other bioactive components derived from serum or RAW264.7 macrophages, which could contribute to the observed effects. Although conditioned media from untreated macrophages was used as a control to account for baseline secretion, the contribution of non-ASC-derived EVs cannot be completely excluded. Future studies using EV-depleted serum and additional characterization approaches will be necessary to further clarify the specific effects of ASC-derived EVs.

Furthermore, EV isolation using a commercial kit (exoEasy Maxi kit) may result in co-isolation of other bioactive components, which could potentially contribute to the observed effects [40,41]. Therefore, the results should be interpreted with caution, and further studies using more refined purification strategies will be necessary to confirm the specific role of EVs.

Finally, EV characterization in this study was based on a limited set of markers. Additional validation using negative and inner EV markers, as recommended by MISEV guidelines, would further strengthen the characterization of the EV population.

In conclusion, our study highlights a critical role of macrophage-mediated inflammatory amplification in uterine smooth muscle activation and demonstrates that ASC-derived EVs effectively suppress this process by targeting NF-κB signaling and downstream contractile responses. These findings provide new insights into the molecular mechanisms underlying inflammation-associated preterm labor and suggest that ASC-EVs may represent a promising cell-free therapeutic strategy for its prevention.

## 4. Materials and Methods

### 4.1. Reagents

Primary antibodies against p44/42 ERK1/2 (#4695), phospho-p44/42 ERK1/2 (#4370), phospho-SAPK/JNK (#9251), phospho-NF-κB p65 (Ser536) (#3033), NF-κB p65 (#8242), phospho-IκBα (Ser32) (#2859), and IκBα (#4812) were obtained from Cell Signaling Technology (Danvers, MA, USA). Lamin B1 antibody (ab133741) was purchased from Abcam (Cambridge, UK). GAPDH (sc-47724) and α-tubulin (sc-8035) antibodies were obtained from Santa Cruz Biotechnology (Santa Cruz, CA, USA). Lipopolysaccharide (LPS; L6529) was purchased from Sigma-Aldrich (St. Louis, MO, USA). RIPA buffer (R2002) was obtained from Biosesang (Seoul, Republic of Korea). Resatorvid (TAK-242; S7455) was purchased from Selleck Chemicals (Houston, TX, USA). Protease and phosphatase inhibitor cocktail (11697498001) was obtained from Roche (Indianapolis, IN, USA).

### 4.2. Cell Culture

Human uterine smooth muscle cells (HUtSMCs) were obtained from PromoCell (C-12575, Heidelberg, Germany) and cultured in Smooth Muscle Cell Growth Medium 2 (C-22062, PromoCell (Heidelberg, Germany)) at 37 °C in a humidified atmosphere with 5% CO_2_. Cells were used between passages 3–5. RAW264.7 murine macrophages were cultured in Dulbecco’s modified Eagle’s medium (DMEM) high-glucose (L0103-500, Biowest, Nuaillé, France) supplemented with 10% fetal bovine serum (FBS; Gibco, Thermo Fisher Scientific, Waltham, MA, USA; A5256701) and 0.1% gentamicin (Gibco, Thermo Fisher Scientific, Waltham, MA, USA; Cat# 15750-060) under the same conditions.

### 4.3. Isolation and Purification of ASC-Derived EVs

Adipose-derived stem cells (ASCs; passage 2) were obtained from PromoCell (C-12977, Heidelberg, Germany) and expanded to passage 4 using Mesenchymal Stem Cell Growth Medium 2 (C-28009, PromoCell, Heidelberg, Germany). Cells were subsequently cultured in MEM Alpha + GlutaMAX™ (Gibco, Thermo Fisher Scientific, Waltham, MA, USA; Cat# 32561-037) supplemented with 10% FBS and 0.1% gentamicin. For EVs collection, ASCs were seeded at a density of 2.36 × 10^6^ cells per 150 mm dish, washed three times with Dulbecco’s phosphate-buffered saline (dPBS; 1×, pH 7.4; Biowest, Cat# L0615), and cultured in serum-free medium for 3 days. Conditioned media (CM) were collected (approximately 12 mL per 150 mm dish), centrifuged at 2000 rpm for 3 min, and filtered through a 0.8 μm filter to remove cell debris while minimizing EV loss, according to the manufacturer’s protocol. EVs were isolated using the exoEasy Maxi kit (Qiagen, Hilden, Germany; Cat# 76064) according to the manufacturer’s instructions and stored at −80 °C until use. For functional experiments, EVs were applied at a standardized concentration (3.8 × 10^10^ particles/mL), and equal amounts were used across all experimental conditions. When different culture vessels were used, EV amounts were adjusted based on the culture surface area to ensure consistent dosing per unit area. EVs were isolated from at least three independent ASC cultures (biological replicates). All experiments were performed in at least three independent experiments, with technical replicates where applicable.

### 4.4. Transmission Electron Microscopy (TEM)

Isolated EVs were fixed in 2% paraformaldehyde for 2 min at room temperature. A 10 μL aliquot of the EV suspension was loaded onto formvar/carbon-coated grids (TED PELLA, Redding, CA, USA), allowed to adsorb, and excess liquid was removed using filter paper. The grids were then stained with 2% uranyl acetate and examined using a transmission electron microscope(JEM1011, JEOL, Tokyo, Japan) at 80 kV.

### 4.5. Nanoparticle Tracking Analysis (NTA)

EVs size distribution and concentration were measured using a ZetaView system (Particle Metrix, Germany). Samples were diluted in dPBS to achieve 150–200 particles/frame. It was measured and analyzed at the set of parameters: temperature of 23 °C, sensitivity of 80, shutter speed of 100, and video frame rate of 30 frames per second. Data were processed using ZetaView software (version 8.06.01).

### 4.6. Preparation of Conditioned Media from RAW264.7 Macrophages

RAW264.7 cells were seeded to ~80% confluency and serum-starved in DMEM containing 0.1% FBS for 8 h. Cells were then stimulated with LPS (10, 50, or 100 ng/mL) for 20 h. Culture supernatants were collected, centrifuged at 1500 rpm for 3 min to remove debris, and used as conditioned media (CM).

### 4.7. Treatment of CM from RAW264.7 Macrophages in HUtSMCs

For CM treatment, HUtSMCs were seeded in 60 mm dishes at a density of 2.5 × 10^5^ cells per dish and cultured for 24 h. Cells were then washed twice with dPBS and incubated in DMEM containing 0.1% FBS for 20 h. The collected CM from RAW264.7 cells was mixed with fresh DMEM containing 0.1% FBS and 0.1% gentamicin at a 1:1 ratio (*v*/*v*) and applied to HUtSMCs for the indicated durations. CM derived from untreated RAW264.7 cells was used as a control to account for baseline secretion of macrophage-derived factors. This approach has been widely used to investigate paracrine inflammatory signaling in similar experimental systems [42,43,44].

### 4.8. Western Blot Analysis

Cells were lysed in RIPA buffer containing protease and phosphatase inhibitors. Equal amounts of protein (30 μg) were separated by SDS-PAGE and transferred onto PVDF membranes. Membranes were blocked with 3% BSA and incubated with primary antibodies overnight at 4 °C. Primary antibodies were used at the following dilutions: anti-GAPDH and anti-ERK1/2 (1:3000), and all other primary antibodies (including p-JNK, JNK, p-ERK1/2, p-p65, and IκBα) at 1:1000. After incubation, membranes were treated with HRP-conjugated secondary antibodies. Signals were detected using an enhanced chemiluminescence system (ATTO Corporation, Tokyo, Japan). Band intensities were quantified using ImageJ software (version 1.54p, NIH, Bethesda, MD, USA) and normalized to the corresponding loading controls. Phosphorylation levels of JNK and ERK1/2 were analyzed by Western blotting using phospho-specific antibodies. The intensity of phosphorylated proteins was normalized to total JNK and ERK1/2 levels, respectively.

### 4.9. Immunofluorescence Staining

Cells cultured on coverslips were fixed with 4% paraformaldehyde, blocked with 3% BSA, and incubated with primary antibodies followed by Alexa Fluor 488-conjugated secondary antibodies. Nuclei were stained with DAPI. Nuclear localization of phosphorylated p65 was analyzed using Fiji, a distribution of ImageJ (https://imagej.net/software/fiji/, accessed on 12 March 2026). 

### 4.10. Cytoplasmic and Nuclear Fractionation

Cytoplasmic and nuclear fractions were prepared using the NE-PER Nuclear and Cytoplasmic Extraction Reagents (Thermo Fisher Scientific, Waltham, MA, USA; Cat. No. 78833) according to the manufacturer’s instructions. Briefly, cells were harvested and sequentially treated with cytoplasmic extraction reagents to isolate the cytosolic fraction, followed by nuclear extraction reagents to obtain nuclear proteins. The purity of each fraction was confirmed by Western blot analysis using GAPDH, a-tubulin as a cytoplasmic marker and Lamin B1 as a nuclear marker. Protein levels in each fraction were analyzed by Western blotting using specific antibodies as indicated.

### 4.11. RNA Isolation and Quantitative Real-Time PCR

Total RNA was isolated from HUtSMCs or RAW264.7 macrophages, depending on the experimental setup, using Easy-Blue reagent (Intron Biotechnology, Seongnam, Republic of Korea). Specifically, RNA used for Figure 5 was isolated from RAW264.7 cells, whereas all other experiments were performed using HUtSMCs. cDNA was synthesized using PrimeScript™ RT Master Mix (Takara Bio, Shiga, Japan; Cat# RR036A). Quantitative real-time PCR was performed using TB Green^®^ Premix Ex Taq™ II (Takara, Shiga, Japan; Cat# RR420A) with gene-specific primers (Table 1). Reactions were carried out on a LightCycler^®^ 480 II system (Roche Diagnostics, Basel, Switzerland).

### 4.12. Enzyme-Linked Immunosorbent Assay (ELISA)

IL-6, IL-8, and MCP-1 levels were measured using ELISA kits, including Human IL-6 ELISA Kit (RayBiotech, Peachtree Corners, GA, USA; Cat# ELH-IL6-1), Human IL-8 ELISA Kit (RayBiotech, Peachtree Corners, GA, USA; Cat# ELH-IL8-1), and Human MCP-1 ELISA Kit (RayBiotech, Peachtree Corners, GA, USA; Cat# ELH-MCP1-1), according to the manufacturer’s instructions.

### 4.13. PKH67 Labeling and Cellular Localization of ASC-EVs

EVs were labeled using the PKH67 Green Fluorescent Cell Linker Kit (Sigma-Aldrich, St. Louis, MO, USA; Cat# PKH67GL) according to the manufacturer’s instructions and incubated with HUtSMCs. Fluorescence signals were observed after 24 h using a fluorescence microscope.

### 4.14. Calcium Flux Assay

Intracellular calcium influx was assessed by measuring fluorescence intensity using a Fluo-4 Direct Calcium Assay Kit (Invitrogen, Thermo Fisher Scientific, Waltham, MA, USA; Cat# F10471) according to the manufacturer’s instructions. HUtSMCs (3 × 10^3^ cells/well) were seeded in black clear-bottom 96-well plates and incubated for 24 h. Cells were then serum-starved in DMEM supplemented with 0.1% FBS and 0.1% gentamicin for 16 h prior to treatment. CM from LPS-stimulated RAW264.7 cells was mixed with fresh DMEM (1:1, *v*/*v*) containing 0.1% FBS and 0.1% gentamicin and applied to HUtSMCs for 24 h. Cells were subsequently loaded with Fluo-4 Direct calcium loading solution supplemented with 5 mM probenecid and incubated for 1 h at 37 °C in 5% CO_2_. Fluorescence intensity was measured using an Infinite 200 PRO microplate reader (Tecan, Männedorf, Switzerland) at excitation/emission wavelengths of 494/516 nm. Changes in intracellular calcium levels were expressed as relative fluorescence intensity normalized to baseline values.

### 4.15. Collagen Gel Contraction Assay

Cell contractility was assessed using a collagen-based cell contraction assay kit (CBA-201, Cell Biolabs, Inc., San Diego, CA, USA) according to the manufacturer’s instructions. Briefly, the collagen gel working solution was prepared by mixing collagen solution with 5× DMEM and immediately neutralized. HUtSMCs were harvested and resuspended at a density of 5 × 10^6^ cells/mL, and a collagen lattice was generated by mixing two parts of the cell suspension with eight parts of the collagen gel solution. The cell–collagen mixture (0.5 mL) was dispensed into each well of a 24-well plate and incubated at 37 °C for 1 h to allow gel polymerization. After polymerization, serum-free medium containing ASC EVs was added and incubated for 30 min, followed by the addition of CM from LPS-stimulated RAW264.7 cells. Gels were then gently detached from the well edges to initiate contraction. Changes in gel size were monitored over 24 h, and gel area was quantified using ImageJ software.

### 4.16. Statistical Analysis

All experiments were performed at least three independent times. Data are presented as mean ± SEM. Statistical significance was determined using one-way or two-way ANOVA followed by Bonferroni post hoc tests. Differences were considered significant at *p* < 0.05.

## 5. Conclusions

In summary, this study demonstrates that macrophage-derived inflammatory signals play a central role in activating uterine smooth muscle cells through TLR4-dependent MAPK and NF-κB signaling pathways. ASC-derived EVs effectively suppress this macrophage-mediated inflammatory amplification by inhibiting IκB degradation, reducing nuclear translocation of p65, and attenuating downstream cytokine expression. Importantly, these effects translate into reduced intracellular calcium signaling and contractile activity in HUtSMCs. Collectively, our findings suggest that ASC-derived EVs represent a promising cell-free therapeutic approach for preventing inflammation-associated preterm labor by simultaneously targeting immune-mediated inflammation and uterine contractility.

## Figures and Tables

**Table 1:** Quantitative real-time PCR with gene-specific primers.

Gene Name	F/R	Primer Sequences (5’-3’)
*GAPDH*	Forward	GGA TTT GGT CGT ATT GGG
	Reverse	GGA AGA TGG TGA TGG GAT T
Primer for Inflammation-related genes		
*TSG6*	Forward	TCT GGC AAA TAC AAG CTC ACC
	Reverse	CTG CCC TTA GCC ATC CAT CC
*MIP2*	Forward	GAA CTG CGC TGC CAG TGC TT
	Reverse	CGA TGC GGG GTT GAG ACA AG
*IL6*	Forward	ACT CAC CTC TTC AGA ACG AAT TG
	Reverse	CCA TCT TTG GAA GGT TCA GGT TG
*IL8*	Forward	TTT TGC CAA GGA GTG CTA AAG A
	Reverse	AAC CCT CTG CAC CCA GTT TTC
*MCP-1*	Forward	CCG AGA GGC TGA GAC TAA CC
	Reverse	GGG GCA TTG ATT GCA TCT GG
Primer for uterine contraction-related genes		
*COX-2*	Forward	CGG TGA AAC TCT GGC TAG ACA G
	Reverse	GCA AAC CGT AGA TGC TCA GGG A
*OXTR*	Forward	TCA TCG TGT GCT GGA CGC CTT T
	Reverse	CGT GAA CAG CAT GTA GAT CCA GG
*Cx43*	Forward	GGA GAT GAG CAG TCT GCC TTT C

## Data Availability

The data presented in this study are available from the corresponding author upon reasonable request.

## Supplementary Materials

The following supporting information can be downloaded at: https://www.mdpi.com/article/10.3390/ijms27104273/s1.

## Abbreviations

The following abbreviations are used in this manuscript:

ASC	Adipose-derived stem cells
ASC-EVs	EVs derived from adipose-derived stem cells
BDM	Blebbistatin
CM	Conditioned media
DAPI	4′,6-Diamidino-2-phenylindole
DMEM	Dulbecco’s Modified Eagle’s Medium
ELISA	Enzyme-linked immunosorbent assay
ERK	Extracellular signal-regulated kinase
FBS	Fetal bovine serum
HUtSMCs	Human uterine smooth muscle cells
IL	Interleukin
IκB	Inhibitor of κB
JNK	c-Jun N-terminal kinase
LPS	Lipopolysaccharide
MAPK	Mitogen-activated protein kinase
MCP-1	Monocyte chemoattractant protein-1
MIP	Macrophage inflammatory protein
NF-κB	Nuclear factor kappa B
NTA	Nanoparticle tracking analysis
PCR	Polymerase chain reaction
PKH67	Green fluorescent cell linker dye
qRT-PCR	Quantitative real-time polymerase chain reaction
RAW264.7	Murine macrophage cell line
SEM	Standard error of the mean
SFM	Serum-free media
TAK242	TLR4 inhibitor (Resatorvid)
TEM	Transmission electron microscopy
TLR4	Toll-like receptor 4
TNFα	Tumor necrosis factor-alpha
TSG6	Tumor necrosis factor-inducible gene 6
