# Supplementary material for: ASC-Derived Extracellular Vesicles Suppress Macrophage-Driven Inflammatory Amplification and Contractile Activation of Uterine Smooth Muscle Cells
Source: Int J Mol Sci. 2026 May 11;27(10):4273. doi: 10.3390/ijms27104273 (PMC13206888; doi:10.3390/ijms27104273)
Supplement: Supplementary file 1 [file ijms-27-04273-s001.zip › ijms-4280589-supplementary/Supplementary Figures S1-S3 and Supplementary Methods.pdf]

Supplementary Figures

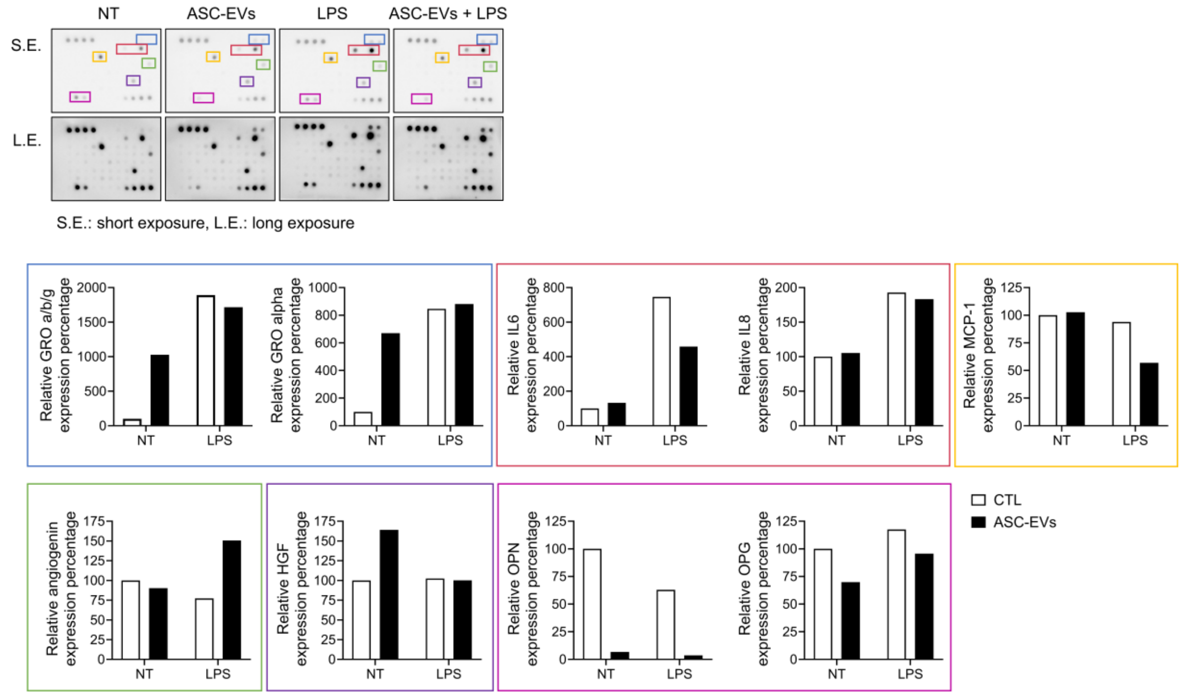

Supplementary Figure S1. Cytokine antibody array analysis of inflammatory mediators.

HUtSMCs were treated with LPS in the presence or absence of ASC-EVs. Representative cytokine array membranes are shown under short and long exposure conditions. LPS stimulation increased the expression of multiple inflammatory mediators, including GROα, IL-6, IL-8, MCP-1, and MIP-2, which were attenuated by ASC-EV pretreatment.

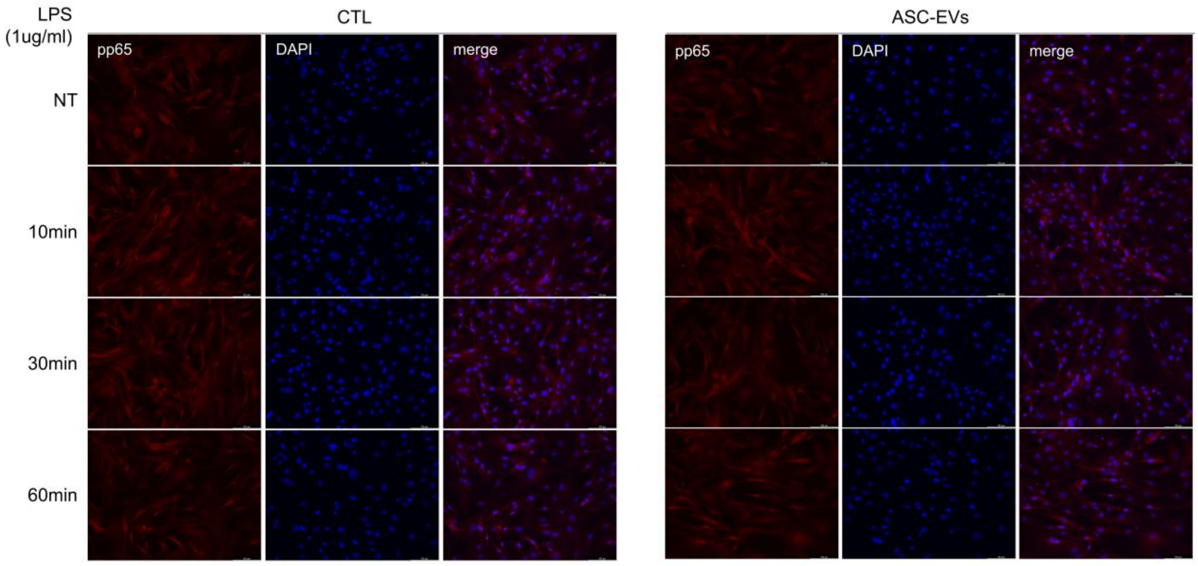

Supplementary Figure S2. Direct LPS stimulation does not induce NF-κB nuclear translocation in HUtSMCs.

HUtSMCs were directly stimulated with LPS (1 μg/mL) for the indicated time points. Immunofluorescence analysis showed no detectable nuclear localization of phosphorylated p65 under these conditions.

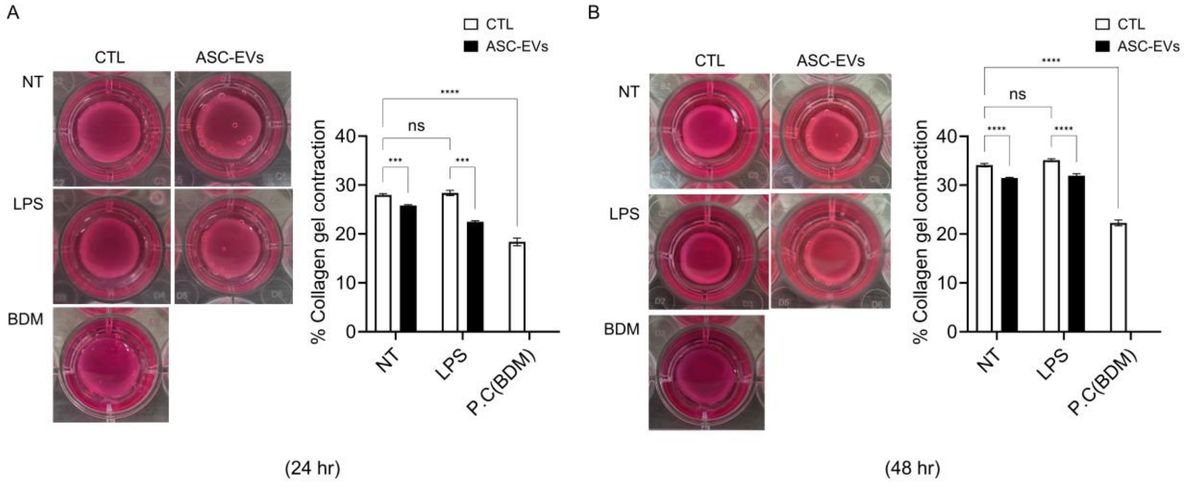

Supplementary Figure S3. Effects of direct LPS stimulation on collagen gel contraction.

Representative images and quantification of collagen gel contraction at 24 h (A) and 48 h (B) following direct LPS stimulation. ASC-EVs pretreatment attenuated gel contraction. Blebbistatin (BDM) was used as a positive control.

Supplementary Methods

Cytokine Antibody Array

Expression of cytokines was analyzed using a human cytokine array C5 (RayBiotech, AAH-CYT-5-4) according to the manufacturer's instructions. Briefly, conditioned media collected from HUtSMCs treated with LPS in the presence or absence of ASC-EVs were applied to the array membranes. Membranes were processed according to the manufacturer's protocol, and chemiluminescent signals were detected. For quantification, dot intensities were analyzed using ImageJ software (NIH, Bethesda, MD, USA). Signal intensities were normalized to the positive control spots on each membrane, and relative cytokine expression levels were calculated accordingly.
